# Supplementary material for: Fighting Antimicrobial Resistance: Development and Implementation of the Ghanaian National Action Plan (2017–2021)
Source: Antibiotics (Basel). 2022 May 3;11(5):613. doi: 10.3390/antibiotics11050613 (PMC9137880; doi:10.3390/antibiotics11050613)
Supplement: Supplementary file 1 [file antibiotics-11-00613-s001.zip › antibiotics-1685709-supplementary.pdf]

Supplement:

Background to emerging research areas on the implementation of the Ghanaian NAP on AMR

(1) Why is the implementation of nationwide AMR surveillance still an uncompleted goal?

During the process of developing the NAP (and even before) there has been a considerable body of research on the prevalence of AMR related to specific substances and in specific locations in Ghana [[1]], including overviews on nationwide surveillance of antibiotic resistance [[2]]. Nevertheless, in the context of the NAP a more precise nation-wide AMR surveillance (continuous monitoring the prevalence of AMR as well as the consumption of anti-microbials) is essential. Ghana did not deliver data for the 2020 and 2021 WHO Global Antimicrobial Resistance and Use Surveillance System (GLASS) Reports [[3]]. We want to assess which aspects of the surveillance system have not been implemented and identify possible obstacles and sources for the delay.

(2) How does cooperation between One Health stakeholders develop and what is the impact of potential conflicts?

The "One Health" concept requires an integral attention towards the use of antibiotics in human health as well as in the livestock sector and the veterinary system, while it also points to the surveillance of resistant pathogens in the environment (waste from hospitals, agriculture and households, in particular in rivers and lakes). Available documents and analyses see One Health as a concerted effort to address conflicts among stakeholders in the fight against fight AMR. Considering experiences in other countries, however, it can be expected that numerous conflicts exist, in particular between growth promotion in the livestock sector and policies to significantly reduce the use of antibiotics in agriculture. Furthermore, the role of different stakeholders in curbing AMR will need more attention. In various papers, it has been pointed out that structures [4]to target AMR and "One Health" approaches need improvement within veterinary services [[5, 6, 7]]. This is also pointed out in a study on "Antimicrobial drug usage and poultry production" [[8]]

(3) To what extent are procurement and distribution of antibiotics regulated and regulations enforced?

The paths of procurement of medicines, legal and illegal, are essential for controlling access to antibiotics. It has to be taken into account that even legal channels of procurement might not necessarily comply with all regulations (lack of surveillance; corruption), that some regulations might be contradictory [[9]] and that counterfeit and substandard medicines frequently enter the supply chain in many countries. This is a crucial factor exacerbating AMR as it can increase overuse through access to antimicrobials without prescription. Furthermore, substandard products can foster resistance and lead to ineffective treatment if there is a lack of active ingredients. There is an interesting study on the use of antibiotics at a market place in Kumasi [[10]], pointing out that women in particular purchase antibiotics from non-pharmacy outlets and market peddlers. More information on the efforts of the Food and Drugs Authority (FDA) should be gained through the interviews.

(4) Are concepts of the NAP transformed into enforceable regulations?

In order to understand governance structures and their shortcomings, we also need to gather more information on the political

processes of transforming the concepts of the NAP into concrete regulations and creating the conditions to enforce them. According to the NAP, the MoH sets up an AMR Secretariat, which convenes quarterly AMR Platform meetings, and other governance links. Furthermore, the role of the Policy Planning, Monitoring and Evaluation Department at the MoH should be analysed. Authors of overview articles on AMR politics in Ghana and because of the pivotal role of the AMR Platform, members of the NPAR should be the important experts to be approached as interview partners for a critical review of governance processes.

(5) How are the financial means for implementing the NAP supposed to be mobilized?

Mobilizing sufficient financial means constitutes an essential aspect of realizing the goals of the NAP. The Financial Plan constitutes one important section of the NAP. There is a very detailed "costing" of all its elements (i.e. of nearly 150 budget entries). Table 2 reproduces the first lines of the budget proposed [11]. It gives an idea of the assumed costs of implementation of the activities listed in the Strategic and the Operational Plan and specifies the sources of funding (see below Section 5.2) The total budget (for 2017 to 2021) is planned to be US$ 21,276,047.93. The Strategic Plan defines timelines for "strategic interventions" and "activities" related to the AMR goals (see Table 1), defined as Strategic Objectives and Sub-Objectives, the Operational Plan determines the lead implementers and the collaborators for each activity. The funding structure for the NAP remains unclear since for the majority of NAP budget items listed for each objective, the sources of funding are indicated as rather generic categories such as: "GOG (Government of Ghana), DPs (Development Partners), Corporate Institutions, NGOs" – i.e., whoever might be ready to pay. In general, no specific ministry budgets or organisations are named.

Table S1. Example of Budget Proposed (Strategic Objective 1, first lines).

Activities	Lead implementer/ Responsibility	Cost per Unit Activity (USD)	factor	freq.	Other Cost	Indicative Budget (USD)	Budget narrative	Source of Funding
**Budget-Strategic Objective 1**						**684,576.04**		
Budget: Sub-objective 1						684,576.04		
5.1.1.1.1. Engage CSOs and the media (as AMR media partners) to educate the public on responsible use of antimicrobials in the spirit of 'One health'	GCNH, VSD, GHS	17,500.00	1	2	0	35,000.00	Budget covers the cost of media training workshops	GOG, DPs, Corporate Institutions, NGOs
5.1.1.1.2. Develop Information, Education and Communication (IE & C) materials for targeted groups in a stratified public education campaign	GHS (Health Promotion Unit), MOFA (ISD), VSD, APD	15,000.00	1	1	40000	55,000.00	Budget covers development of education and communications kit for the public education campaign on antibiotics	GOG, DPs, Corporate Institutions, NGOs
5.1.1.1.3. Educate the public in order to promote the responsible use of antimicrobials among the general population	VSD, GHS, GNDP	70,000.00	1	5	60000	410,000.00	Budget covers media campaign TV programmes, Radio programmes, Social media campaign etc. as well as support for antimicrobial week in November	GOG, DPs, Corporate Institutions, NGOs
5.1.1.1.4. Review the public education campaign for optimized impact	VSD, GHS	7,000.00	1	1	0	7,000.00	Consultancy to review the success and challenges of the public education campaign and make recommendations	GOG, DPs, Corporate Institutions, NGOs

When reviewing the Budget Statement and Economic Policy of the Government of Ghana for the 2019 Financial Year [12], the terms "NAP" or "AMR" or "surveillance" (related to health) do not appear. These

findings are also reflected by a recent study on transparency of AMR NAPs in African countries, which found that budget transparency was very low [13].

The WHO Country level report [14] focusses on the issue of "getting AMR into existing programs" concentrating on the aspect of funding (note the main title: "Resource mobilisation for AMR"). Certainly, there is a good chance to mobilise external funds for many activities which will have to be developed with the implementation of the NAP. Strategies for that are proposed within the WHO report and in some other papers [15] (see the following research question). It will be an important point to discuss with all the experts to be interviewed, how they assess the financial situation concerning their respective activities and the role of the government in this field.

(6) Which contributions can be expected from international partners?

International partners have played an important role for the development and implementation of the NAP including the dimension of finance. From the governance perspective, the AMR Secretariat and the AMR Platform should play a crucial role, but that is not clear from the available documents [16]. Japheth A. Opintan was the Scientific research coordinator of the ADMER project. The role of WHO, FAO, OIE (partners of the Tripartite Collaboration on AMR) and (more recently) also of the UN Environmental Programme (UNEP), both in international cooperation as well as through their Ghanaian offices, should be better understood.

The WHO country level report [17] checks the needs of all key ministry departments and agencies. It refers to existing partnerships with the United States Centres for Disease Control (US CDC) Partnership for Health Care, the Global Fund, the United Stated Agency for International Development (USAID and the Noguchi Memorial Institute (JICA) for AMR surveillance, with "USAID and other partners" in the Institutional Care Division; with the Global Environmental Facility, World Bank, UNEP and Norway in the Environmental Sector. The last part of the report informs about the most important "development partners and donors" (24-30), mostly under the heading "AMR integration opportunities". Only in a few cases finance is explicitly related to AMR policy (UK Department for International Development (DFID), Fleming Fund, FAO: awareness to risks of AMR).

Until 2019, the Fleming Fund Ghana Country Grant [18] has been the only large cooperation project, which supports a comprehensive implementation of the NAP. It has a volume of 2.352 million US$, which amounts to 11.05% of the total budget for the NAP. The grant objectives are summarized in three points (see also: Fleming Fund, website [19]):

- establish a well-functioning One Health governance structure for AMR and AMU surveillance
- establish a government led system of collecting, collating, analysing, reporting and disseminating AMR and AMU data on national and international platforms in alignment with the requirements of the WHO Global Antimicrobial Resistance and Use Surveillance System (GLASS reports).
- strengthen AMR and AMU surveillance in animals.

How further international partners and innovative technology (e.g. in the fields of networking and national coordination) can be mobilized, remains an important question.

(7) How successful has been AMR-related capacity-building in academic institutions?

Increasing capacities of experts in the various fields linked to fighting AMR (medical personnel, biomedical researchers, pharmacists, veterinarians, environmental scientists etc.) constitutes an important element in the implementation of the NAP. This refers primarily to AMR-related teaching, training and research. While progress in research is expressed in the growing number of academic publications on topics linked to AMR, it is difficult to find out from desk review whether these topics have been more strongly embedded in academic education.

(8) Can we observe a growing awareness of AMR in Ghanaian society?

Awareness building for a broad understanding of the threat of AMR is the first goal in the NAP framework; it has been present once a year during the World Antibiotic Awareness Week (WAAW), but not very visible in the media throughout the rest of the year. One article from a research group at the University of California at Irvine [20] (discusses this topic and found that awareness of AMR is not very high in the Ghanaian society. We were able to identify a few civil society groups with a focus on health in Ghana, in particular the CSO Hope for Future Generations and the Coalition of NGOs in Health. More information is needed on the strategies to improve general public awareness in Ghana, e.g. through specific events directed at the general public (and the publicity for these events), the role of the media in Ghana, in particular radio and TV. As part of the desk review we perused the internet information services "Savannah News", "Citi Newsroom", and "Ghana Web" with regard to AMR. We were not able to identify many news items on AMR between January 2017 and July 2019. Those identified do not refer to local AMR politics but to WHO statements and activities in this field. The most visible activity is the WAAW, which has been marked by increasing activities in Ghana since its international launch by WHO in 2015. Interviews with journalists and other media specialists would be helpful.

(9) What is the role of Ghana in international health?

Ghana is frequently considered a leading country on the African continent for handling AMR. This is due to its presence in academic and political events on this matter (in particular, its role in the Global Call to Action on AMR) and the Ghanaian approach to the formation of a national working group on antibiotic resistance, leading to the NAP (the NPAR) [21]. We want to further explore the role of Ghana in international health from different viewpoints through expert interviews: (1) Self-image of Ghana among the general population, which could have positive (e.g. contributing to political stability) or negative impacts (e.g. tendency to accept deficits). (2) Higher standing in the international health community, which give Ghanaian scientists a more important role in shaping and benefitting from international networking. (3) Access to international cooperation and finance. These issues require information from different respondents.

(10) What about monitoring and evaluation of the NAP (2017-21) and a possible NAP 2?

As early as January 2017, the WHO presented a detailed concept for Monitoring and Evaluation of the Global Action Plan on Antimicrobial Resistance [22]. Available data would allow to present some information on some of the output indicators proposed in the document, but so far no comprehensive Monitoring and Evaluation report on the Ghanaian NAP has been presented. There are other signs indicating that the implementation of the NAP on AMR is not pursued with particular urgency.

In August 2019 a short press release titled "Ghana's National Action Plan on AMR on Course" was published by the Ghanaian government, stating among others "the launch of the Policy and NAP was followed by the *first* [emphasis, W.H.] Interministerial Committee meeting on March 21, 2019 to sensitize implementing ministries and agencies on AMR country actions and, specifically the activities of the Fleming Fund Project". Thus, the "highest decision-making body with oversight responsibility for the implementation of the interventions on AMR" started one year after the Ghanaian president had officially launched the NAP (which formally had already started in 2017) [[23]]. Furthermore, in 2018 a discourse was started to develop a National Action Plan for Health Security (NAPHS)[[24]]. The Budget Statement for 2020 refers to the launch and implementation of the NAPHS (article 1028) [[25]], while there is no reference to the NAP on AMR.

The NAP is designed for the period 2017 until 2021. Through online desk review, no reference to any discussion on a possible prolongation of the plan (or a follow-up strategy) could be found.

All issues (1-10) have been addressed in the expert interviews with stakeholders that are involved in the governance of AMR in Ghana. The results of the interviews are presented below, followed by a general analysis of all results.

---

[1] García-Vello, P.; González-Zorn,B.; Setsoafia Saba, C.K. Antibiotic resistance patterns in human, animal, food and environmental isolates in Ghana: a review, *Pan African Medical Journal* **2020**;35:37. Available online: https://www.ncbi.nlm.nih.gov/pmc/articles/PMC7245977/pdf/PAMJ-35-37.pdf (accessed on 15/03/2022).

[2] Opintan, J.A.; Newman, M.J.; Arhin, R.E.; Donkor, E.S.; Gyansa-Lutterodt, M.; Mills-Pappoe, W. Laboratory-based nationwide surveillance of antimicrobial resistance in Ghana. *Infect Drug Resist.* **2015** Nov 18;8:379-89. doi: 10.2147/IDR.S88725. eCollection 2015. Available online: https://pubmed.ncbi.nlm.nih.gov/26604806/ (accessed on 15/03/2022).

[3] WHO, Global Antimicrobial Resistance and Use Surveillance System (GLASS) Report 2021. Geneva. Available online: https://apps.who.int/iris/rest/bitstreams/1350455/retrieve (accessed on 15/03/2022).

[4] cancelled

[5] Yevutsey, S. K.; Buabeng, K.O.; Aikins, M.; Anto, B.P.: Biritwum, R.B.; Frimodt-Møller, N.; Gyansa-Lutterodt, M. Situational analysis on antibiotic use and resistance in Ghana: policy and regulation, BMC Public Health 2017; 17:896.

[6] WHO (2018). Resource mobilisation for antimicrobial resistance (AMR): Getting AMR into plans and budgets of government and development partners. Ghana country level report. Available online: https://www.who.int/antimicrobial-resistance/national-action-plans/Ghana-AMR-integration-report-WHO-June-2018.pdf?ua=1 (accessed on 15/03/2022).

[7] The Fleming Fund (2018): Terms of Reference for Request for Proposals. Ghana First Country Grant. Available online: https://www.flemingfund.org/wp-content/uploads/c9321a9a51d977b11bec3e95c8fb9438.pdf (accessed on 16/03/2022.

[8] Johnson, S.; Bugyei K.; Nortey, P.;Tasiame,W. Antimicrobial drug usage and poultry production: case study in Ghana. *Animal Production Science,* published December **2017.** Available online: https://www.publish.csiro.au/an/AN16832 (accessed on 16/03/2022).

[9] Yevutsey, S. K.; Buabeng, K.O.; Aikins, M.; Anto, B.P.: Biritwum, R.B.; Frimodt-Møller, N.; Gyansa-Lutterodt, M. Situational analysis on antibiotic use and resistance in Ghana: policy and regulation, BMC Public Health 2017; 17:896.

[10] Hitch, G.; Danquah, C.A.; Owusu-Ofori, A.; Agyarko-Poku, T.; Manfrin, A.; Owusu-Dabo, E.; Buabeng, K.O. Public perceptions on the use of antibiotics at a market place in Kumasi, Ghana: A cross-

sectional study. *Global Journal of Medicine& Public Health* **2020**; 9 (1). Available online: http://clok.uclan.ac.uk/35806/ (accessed on 16/03/2022).

[11] Republic of Ghana, Ministry of Health , Ministry of Food and Agriculture, Ministry of Environment, Science Technology and Innovation, Ministry of Fisheries and Aquaculture Development. Ghana National Action Plan for Antimicrobial Use and Resistance. 2017-2021, Accra, 2017.

[12] Republic of Ghana (2019): The Budget Statement and Economic Policy of the Government of Ghana for the 2019 Financial Year. Available online: https://www.mofep.gov.gh/sites/default/files/budget-statements/2019-Budget-Statement-and-Economic-Policy.pdf (accessed on 16/03/2022).

[13] Harant, A. Assessing transparency and accountability of national action plans on antimicrobial resistance in 15 African countries. Antimicrobial Resistance and Infection Control 2022, 11:15. Available online: https://doi.org/10.1186/s13756-021-01040-4 (accessed on 15/03/2022).

[14] WHO (2018). Resource mobilisation for antimicrobial resistance (AMR): Getting AMR into plans and budgets of government and development partners. Ghana country level report. Available online: https://www.who.int/antimicrobial-resistance/national-action-plans/Ghana-AMR-integration-report-WHO-June-2018.pdf?ua=1 (accessed on 15/03/2022).

[15] Opintan, J. Leveraging donor support to develop a national antimicrobial resistance policy and action plan: Ghana's success story, *Afr J Lab Med. 2018;7(2), a825.* Available online: *https://doi.org/10.4102/ajlm.v7i2.825* (accessed on 16/03/2022).

[16] See [xv]

[17] See [xiv]

[18] The Fleming Fund (2018): Terms of Reference for Request for Proposals. Ghana First Country Grant. Available online: https://www.flemingfund.org/wp-content/uploads/c9321a9a51d977b11bec3e95c8fb9438.pdf (accessed on 16/03/2022 Fleming Fund Country Grant

[19] The Fleming Fund (website): Our Activities: Governance. Support Strong National AMR Governance. Available online: https://www.flemingfund.org/wp-content/uploads/debcf5faea0903a502c05326a4ac3c1c.pdf (accessed on 16/03/2022).

[20] Jimah,T.; Fenny, A.P.; Ogunseiten, O. Antibiotics stewardship in Ghana: a cross-sectional study of public knowledge, attitudes, and practices among communities, *One Health Outlook* **2020**; 2, 12.

[21] ReAct. About us. Our story. (section: 2015: ReAct Africa). Available online: https://www.reactgroup.org/about-us/our-story// (accessed on 16/03/2022).

[22] WHO. Monitoring and Evaluation of the Global Action Plan on Antimicrobial Resistance. Regional Expert Consultation on Monitoring and Evaluation of AMR Interventions. Available online https://www.paho.org/hq/dmdocuments/2017/2017-cha-walford-global-dc.pdf (accessed on 16/03/2022).

[23] Zaney, G.D. Ghana's National Action Plan On AMR Implementation On Course? but It Requires Support From All. *GH Headlines*, AllAfrica News Ghana, 27th August **2019**. Available online: http://www.ghheadlines.com/agency/all-africanews--ghana/20190827/130866936/ghanas-national-action-plan-on-amr-implementation-on-coursebut-it-requires-support-from-all (accessed on 16/03/2022).

[24] WHO Africa. Ghana advances towards developing a National Action Plan for Health Security. 06 July 2018. Available at: https://www.afro.who.int/news/ghana-advances-towards-developing-national-action-plan-health-security (accessed on 18/03/2022).

[25] Republic of Ghana (2020): The Budget Statement and Economic Policy of the Government of Ghana for the 2020 Financial Year, Accra, Available online: https://www.mofep.gov.gh/sites/default/files/budget-statements/2020-Budget-Statement-and-Economic-Policy_v3.pdf (accessed on 16/03/2022).
